# Human ventricular activation sequence and the simulation of the electrocardiographic QRS complex and its variability in healthy and intraventricular block conditions

**DOI:** 10.1093/europace/euw346

**Published:** 2016-12-23

**Authors:** Louie Cardone-Noott, Alfonso Bueno-Orovio, Ana Mincholé, Nejib Zemzemi, Blanca Rodriguez

**Affiliations:** 1 Department of Computer Science and British Heart Foundation Centre of Research Excellence, University of Oxford, Oxford, UK; 2 INRIA Bordeaux Sud-Ouest, 200 avenue de la vieille tour, Talence Cedex, France; 3 IHU Liryc, Electrophysiology and Heart Modeling Institute, foundation Bordeaux Université, Pessac Bordeaux, France

**Keywords:** Electrocardiogram, QRS complex, Activation sequence, Variability, Computer modelling and simulation

## Abstract

**Aims:**

To investigate how variability in activation sequence and passive conduction properties translates into clinical variability in QRS biomarkers, and gain novel physiological knowledge on the information contained in the human QRS complex.

**Methods and results:**

Multiscale bidomain simulations using a detailed heart-torso human anatomical model are performed to investigate the impact of activation sequence characteristics on clinical QRS biomarkers. Activation sequences are built and validated against experimentally-derived *ex vivo* and *in vivo* human activation data. R-peak amplitude exhibits the largest variability in terms of QRS morphology, due to its simultaneous modulation by activation sequence speed, myocardial intracellular and extracellular conductivities, and propagation through the human torso. QRS width, however, is regulated by endocardial activation speed and intracellular myocardial conductivities, whereas QR intervals are only affected by the endocardial activation profile. Variability in the apico-basal location of activation sites on the anterior and posterior left ventricular wall is associated with S-wave progression in limb and precordial leads, respectively, and occasional notched QRS complexes in precordial derivations. Variability in the number of early activation sites successfully reproduces pathological abnormalities of the human conduction system in the QRS complex.

**Conclusion:**

Variability in activation sequence and passive conduction properties captures and explains a large part of the clinical variability observed in the human QRS complex. Our physiological insights allow for a deeper interpretation of human QRS biomarkers in terms of QRS morphology and location of early endocardial activation sites. This might be used to attain a better patient-specific knowledge of activation sequence from routine body-surface electrocardiograms.

**What’s New?**

- Multiscale human heart-torso models with anatomical and functional detail based on multimodal human activation data are presented for investigations on variability in clinical QRS biomarkers.
- Activation sequence, myocardial conductivities and torso propagation have distinct effects in modulating R-peak amplitude, QRS width and QR interval.
- Variability in the anatomical location of left ventricular activation sites is associated with S-wave progression in limb and precordial leads, and notched QRS complexes in precordial derivations.
- Variability in the number of early activation sites replicates the clinical QRS manifestations of all hemiblocks and bundle branch blocks of the human conduction system.
- The tools presented here advance the field of computational cardiac electrophysiology towards more predictive technologies for risk assessment at the population level, as well as for the better understanding of clinical ECG biomarkers of disease.

## Introduction 

Since its invention by Einthoven at the beginning of the 20th century, the body-surface electrocardiogram (ECG) remains as the most extensively used clinical tool for the non-invasive diagnosis of cardiac disorders. A specific challenge is to discriminate between the effects on the ECG biomarkers of the different components of the ventricular activation and repolarization sequences (involving simultaneous transmural, apico-basal, posterior-anterior, and inter-ventricular propagation), and/or the contribution of concurrent pathological states. This hampers our ability to effectively extract the maximum information about the heart from the ECG. 

In this context, multiscale human heart models are powerful platforms to integrate electrophysiological and structural information from the ionic to the whole organ levels, and to differentiate between key factors determining ECG biomarkers in disease^1^ or under pharmacological action.^2^ Such an improved knowledge may further aid in the development of more selective biomarkers for specific diseased conditions or drug-induced risk stratification.^3^ Most computational studies to date, however, have mainly concentrated on replicating the repolarization sequence of the human ventricles and placed less attention on the activation sequence, often resulting in poorly-recovered QRS complex morphology and lead polarities (see for example^1^^,^^2^) This highlights the need for accurate representations of the human activation sequence in ECG simulations, due to its direct influence in determining repolarization, and to properly address the computational study of cardiac conduction disorders.

In this work, we investigate the effect of variability in characteristics of the human ventricular activation sequence in the simulated QRS complex using a human torso-heart bidomain model. The simulated activation sequences are built and validated using experimentally-derived human activation data, ranging from *exvivo* microelectrode recordings, non-invasive *invivo* electromechanical wave and electrocardiographic imaging, up to the body-surface ECG. Quantitative investigations on variability in activation sequence and passive conduction properties are then presented to gain novel insights on the impact of tissue-level propagation in clinical QRS biomarkers, as well as to demonstrate the feasibility of our approach to model pathophysiology of the human conduction system. Our results can therefore have important implications in advancing the realistic modelling of cardiac conduction under healthy and diseased conditions, and aid in unravelling the role of variability in modulating response to therapy at the population level.

## Methods

### Experimental data

**Figure 1:**
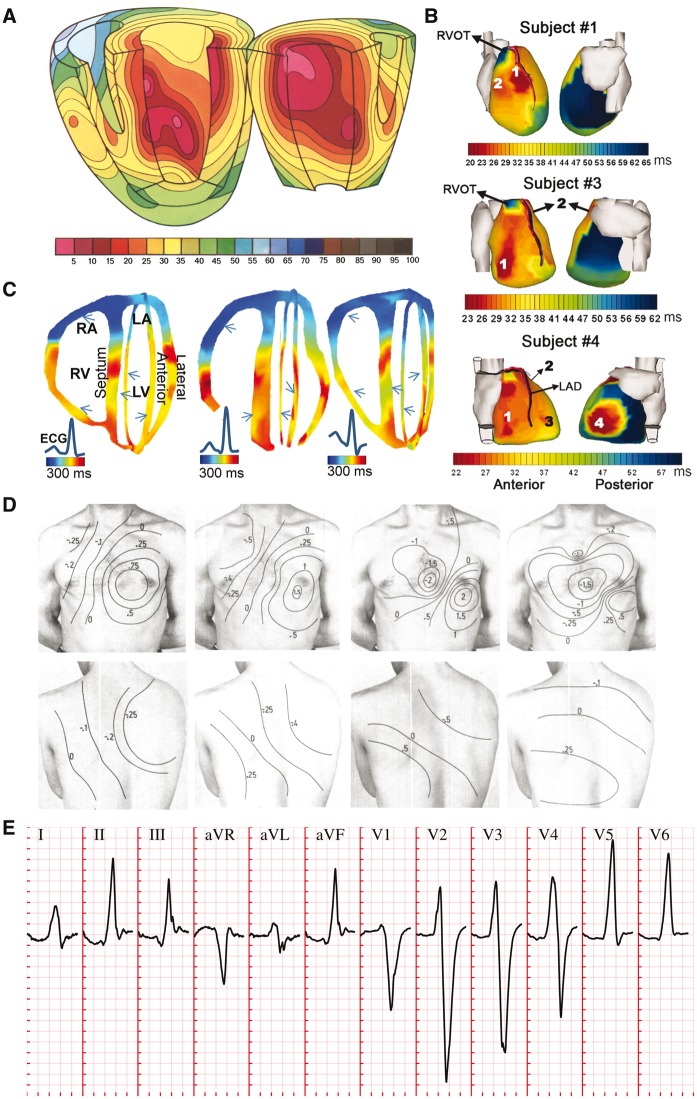
Non-diseased human ventricular activation data considered in this study. (*A*) *Ex vivo* microelectrode recordings of activation sequence in a healthy subject. (*B*) Non-invasive *in vivo* electromechanic wave imaging isochrones of three normal subjects. Arrows indicate ventricular sites of early activation. (*C*) Non-invasive *in vivo* electrocardiographic imaging reconstruction of epicardial activation sequence in three healthy subjects. (*D*) Body surface potential maps in a healthy subject during progression of ventricular activation. (*E*) QRS complexes in the 12-lead ECG of a representative healthy individuals of the PTB Diagnostic ECG Database^8^ (subject 198). Clinical ECG grid resolution: 40 ms/0.1 mV. Panels A–D reproduced with permission from Durrer *et al.*,^4^ Provost *et al.*,^5^ Ramanathan *et al.*^6^ and Taccardi,^7^ respectively.

The present investigations build on three main sources of human experimental data. Firstly, *ventricular activation sequences* were based on *exvivo* microelectrode recordings by Durrer *et al.*^4^ (*Figure **1**A*), *invivo* electromechanical wave imaging by Provost *et al.*^5^ (*Figure **1**B*), and *invivo* epicardial activation sequences as reported by Ramanathan *et al.*^6^ through non-invasive electrocardiographic imaging (*Figure **1**C*). Secondly, distributions of *body-surface potentials* (BSPs) during ventricular activation in healthy conditions were analysed from Taccardi *et al.*^7^ (*Figure **1**D*). Thirdly, 12-lead *body-surface ECG* signals (*Figure **1**E*) in healthy and bundle branch block conditions were obtained from the PTB Diagnostic ECG Database,^8^ freely available in Physionet (www.physionet.org), whilst hemiblock ECGs (not present in the PTB Diagnostic ECG Database) were analysed from Elizari *et al.*^9^

### Anatomical heart-torso model

A human biventricular mesh, embedded in a torso volume containing lung and bone regions, was generated from computer tomography images as described in Supplementary Methods. Virtual electrodes were positioned on the torso at standard electrode locations for the calculation of the 12-lead ECG (Supplementary material online, *Figure S1*).

### Electrophysiological and tissue model

Computer simulations were conducted using the fully coupled heart-torso bidomain equations, which are the gold-standard for the description of cardiac electrical propagation, including detailed description of human ventricular cellular electrophysiology. Myocardial and torso conductivities were based on the literature, as detailed in Supplementary Methods, together with specifications on our numerical solver. Research materials are available upon request.

### Activation system models

As in Keller *et al.*^10^ and due to the variability and lack of high-resolution data on which to build anatomically-detailed models of the human *free-running Purkinje network*, we assume that its branches couple with and excite the endocardial layer at several sites of earliest activation (root points) from which excitation quickly progresses. We base the positions of these root points on the notion of the human trifascicular conduction system^11^ (left anterior-superior, left posterior-inferior and right lateral fascicular branches). To model the tightly-packed *endocardial Purkinje network*, pairwise distances between all endocardial surface nodes were pre-processed using Dijkstra's algorithm. At runtime, each endocardial surface node is assigned a stimulus time proportional to the distance to its closest root node. The constant of proportionality allows a straightforward control of conduction velocity in the abstracted endocardial Purkinje network. This was adjusted to yield a distribution of endocardial activation times in accordance with the reported *exvivo* microelectrode recordings by Durrer *et al.*^4^

## Results

### Model construction and validation

Human ventricular activation data exhibit a high level of inter-subject variability in the sites of earliest activation and epicardial breakthrough, as illustrated in the multi-modality data comparison summarized in *Figure **1*. The following commonalities were however, identified across the studied human datasets: (i) activation within the left ventricle (LV) usually begins on the basal anterior paraseptal, the mid-septum and the posterior apex regions, and then progresses transmurally (*Figure **1**A and B*); (ii) earliest activation in the free wall of the right ventricle (RV) occurs ‘near the insertion of the anterior papillary muscle’,^4^ the position of which is known to be highly variable (*Figure **1**B and C*); and (iii) activation of the RV epicardium usually occurs before that of the LV epicardium (*Figure **1**B and C*).

**Figure 2:**
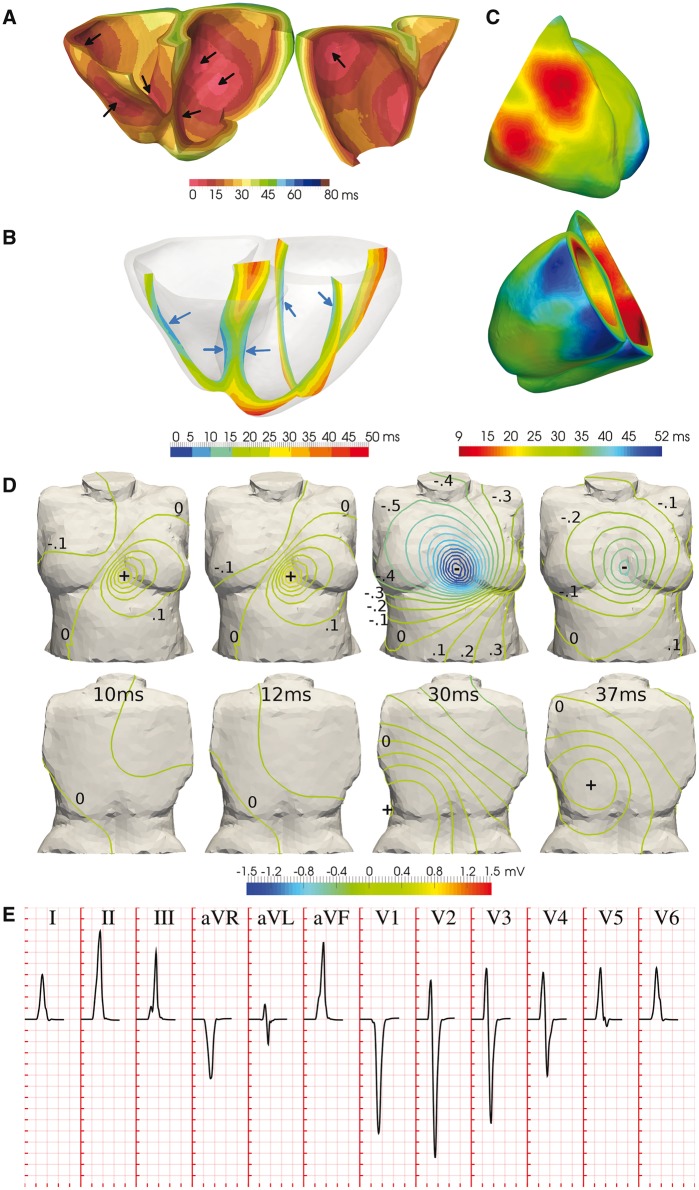
Simulation results of the healthy human ventricular activation sequence. (*A*) Volumetric isochrones of ventricular activation times. Arrows indicate activation system root points. (*B*) Cross-sectional slices of ventricular activation times. Arrows indicate ventricular sites of early activation. (*C*) Anterior and posterior views of epicardial activation times. Different colour maps are shown in Panels A–C to facilitate the comparison with experimental data in Figure 1. (*D*) Isopotential body surface potential maps during progression of ventricular activation. (*E*) QRS complexes in the simulated 12-lead ECG. Simulated ECG grid resolution: 40 ms/0.1 mV.

Based on the above, we designed a baseline configuration of the human activation sequence (see *Figure **2**A*, black arrows) consisting of four LV earliest activation sites (LV mid septum, LV basal anterior paraseptal, and two LV mid-posterior) and three in the RV (RV mid septum, two RV free wall). Such a configuration allows for a multiscale investigation (up to the body-surface ECG) of inter-subject variability in activation speed and passive tissue conductivities, as well as in the position and number of earliest activation sites, without favouring a specific data modality in our analysis.

Simulated ventricular activation times for the baseline configuration are presented in *Figure **2**A*. The LV endocardial surface is fully activated within 30 ms, transmural propagation occurs in 35 ms in the LV basal free wall, with transseptal propagation of 25 ms. This is in accordance with the *exvivo* microelectrode recordings by Durrer *et al.*^4^ (*Figure **1**A*), reporting 35 ms in endocardial LV activation, and 25 and 35 ms in transseptal and transmural propagation, respectively. Slightly shorter activation times are obtained in the RV base in simulations, possibly due to uncertainties in the atria-ventricular segmentation.

Cross-sectional slices of ventricular activation times are shown in *Figure **2**B* for comparison with *invivo* electromechanical wave imaging data by Provost *et al.*^5^ (*Figure **1**B*), indicating agreement in the location of earliest and latest activation sites. Specifically, early activation occurred on either side of the septum, RV free wall, and anterior and posterior LV paraseptal sites. Latest activation took place in the basal septum, basal lateral LV wall, and apex.

Anterior and posterior views of epicardial activation times are provided in *Figure **2**C* for ease of comparison with the *invivo* electrocardiographic imaging data of Ramanathan *et al.*^6^ Simulation results are in close agreement with Subjects 1 and 3 in *Figure **1**C*, with earliest breakthrough in the upper paraseptal anterior RV, apical LV activation 20 ms later, and basal LV activation 28 ms after that. The activation of the RV epicardium completed in 30 ms, compared to 25 ms in healthy subjects. The LV epicardial breakthrough occurred about 15 ms after the RV breakthrough, consistent with both Subjects 1 and 3. The pattern of LV epicardial activation is well reproduced due to the lateral basal LV root point (leading to the epicardial breakthrough on the LV anterior paraseptal region) and the thickening of the LV free wall from apex to base (which generates activation times that lengthen from apex to base).

*Figure* *2* *D* shows isopotential maps of BSPs, exhibiting the same spatiotemporal evolution during ventricular activation as those reported in healthy subjects by Taccardi *et al.*^7^ (*Figure **1**D*). Initially a local maximum appears on the chest due to the first RV epicardial breakthrough (10 ms). Once the activation of the RV is completed, this is replaced by a negative region. The positive pole moves leftwards and downwards as activation progresses transmurally through the LV. This forms the characteristic dipole pattern on the front of the chest with a zero isopotential between the lower-right flank and the left shoulder (20–30 ms). The maximum is finally drawn towards the latest area to activate, the posterior paraseptal LV (40 ms).

As a consequence of the distributions of BSPs described above, a frontal plane QRS axis (average of global ventricular depolarization) between −30° and +90° is considered to be normal, where 0° is leftwards in the transverse plane. This determines the limb (I, II, III) and augmented (aVR, aVL, aVF) leads, and yields negative precordial leads in V1, positive in V4–V6, with R-wave progression in between, as illustrated in *Figure **1**E* for a representative healthy subject. For comparison, *Figure **2**E* illustrates the simulated QRS complexes associated with our baseline activation sequence, exhibiting a QRS width of 70 ms within the normal range (60–100 ms) and a QRS axis of 60°, both within the healthy range, and typical lead polarities in all limb, augmented limb and precordial leads, as well as R-wave progression in the latter.

### Variability in subendocardial activation speed and myocardial conductivities modulate QRS width and amplitude

Detailed simulation studies were conducted to investigate how variability in the different determinants of distributions of BSPs modulates the human QRS complex. Whilst the extent of this variability in the human population is difficult to determine,^12^ we hypothesize that variability of ±50% in activation sequence and tissue properties encapsulates a major fraction of the observed clinical variability in the healthy human QRS complex. Changes on baseline QRS biomarkers are hence presented in the following sections (averages reported across all leads) for these bounds of plausible physiological variability. In fact, this could be a modest estimate of total variability across the population, since variability bounds from 2 to 12-fold have been reported in tissue conductivities in human,^13^ including blood (×2.3), lungs (×3.4), fat (×5.5), soft muscle (×7.5), heart (×9.0) and bone (×12).

**Figure 3:**
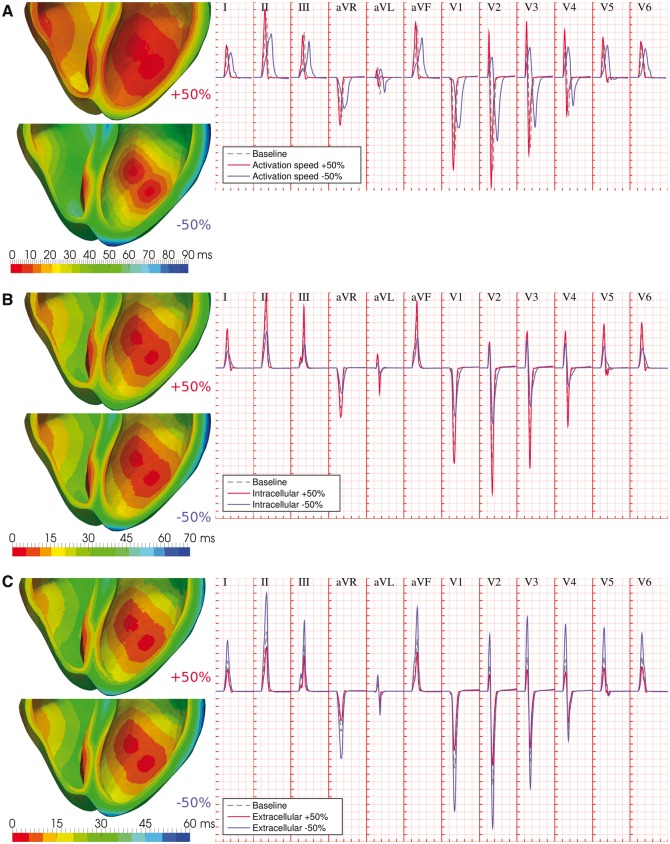
Variability in activation speed and myocardial conductivities modulate QRS width and amplitude. (*A*) Variability in activation speed impacts the ventricular activation sequence, affecting R-wave times-to-peak, R-wave amplitudes, and QRS width. (*B*) Variability in intracellular myocardial conductivities influences the transmural dispersion of ventricular activation times, yielding scaled R-wave amplitudes and wider and less-symmetric QRS complexes under conditions of decreased intracellular coupling. (*C*) Variability in extracellular myocardial conductivity yields a scaling effects on QRS amplitudes. In all cases, dashed grey ECG traces represent baseline conditions, whereas solid red and blue ECG traces represent conditions of increased and decreased myocardial parameters, respectively. Simulated ECG grid resolutions: 40 ms/0.1 mV.

Variability in endocardial activation speed directly impacts the full ventricular activation sequence (*Figure **3**A*, left panel), affecting the width and amplitude of the reconstructed QRS complexes. Increased activation speeds (*Figure **3**A*, solid red traces) resulted in earlier R-wave times-to-peak, shorter QRS widths, and increased R-wave amplitudes (75 ± 3%, 90 ± 4%, and 125 ± 30% of baseline, respectively). Decreased activation speeds had the opposite effects (*Figure **3**A*, solid blue traces), exhibiting protracted R-wave times-to-peak, wider QRS widths, together with decreased R-wave amplitudes (178 ± 7%, 182 ± 12%, and 83 ± 8% of baseline, respectively).

Variability in intracellular myocardial conductivities (as modelled by globally scaled conductivity tensors) substantially influences the transmural dispersion of ventricular activation times (*Figure **3**B*, left panel). This yielded a predominant scaling effect on R-wave amplitudes (121 ± 13% and 65 ± 12% of baseline for increased/decreased intracellular conductivities, respectively), with a smaller impact on times to R-peak (95 ± 3% and 105 ± 3%, respectively). Wider QRS widths (127 ± 7%) and less symmetric QRS complexes were also associated with conditions of decreased intracellular coupling, in particular in the precordial leads (*Figure **3**B*, solid blue traces).

On the other hand, variability in extracellular myocardial conductivities has a purely scaling role on the QRS complex, with negligible effects on the ventricular activation sequence (*Figure **3**C*). Across all leads, peak R-wave amplitudes were 69 ± 6% and 183 ± 25% of baseline for a 50% increase and decrease in extracellular conductivity tensors, respectively. Times to R-peak were within 2% in all cases, with negligible changes to QRS widths.

The observed effects can be explained by recalling a simplified core-conductor model of electrical propagation in myocardial fibers,^14^ with extracellular potentials given by

$$\phi_{e}(t,\;x)\propto \frac{\sigma_{i}}{\sigma_{e}}\int_{\Omega }^{}\nabla \phi_{i}(t)\cdot \nabla \left(\frac{1}{r}\right)d\Omega ,$$

where *ϕ_e_* is the extracellular potential at time *t* and body surface point **x**, *σ_i_* and *σ_e_* are the intracellular and extracellular conductivities, and the integral represents the sum of the contribution of all gradients in intracellular potentials (∇*ϕ_i_*) in the volume of the heart (Ω), weighted by their respective distances to the measure point (***r***). Changes in activation speed therefore affect the time course of endocardial and transmural gradients in intracellular potentials, hastening or protracting ventricular depolarization as reflected in the QRS width and times to R-peak, while their spatial distribution (more spread gradients for larger activation speeds) impacts R-wave amplitudes (*Figure **3**A*). In contrast, variability in intracellular conductivities under the imposition of the same endocardial activation sequence only modulates the transmural propagation of intracellular potentials, hence impacting the terminal part of the QRS complex with moderate influence on times to R-peak, together with a proportional scaling of extracellular potentials (*Figure **3**B*). Correspondingly, extracellular potentials are inversely proportional to extracellular conductivities, modulating R-wave amplitudes with negligible effects on intracellular gradients (*Figure **3**C*). Changes in QRS width by myocardial conductivities can also be interpreted by considering the effective conductivity tensor, *σ_m_* = *σ_i_σ_e_*/(*σ_i _*+ *σ_e_*), and its impact on myocardial propagation speed, given by the square root of the quotient between altered and baseline effective conductivities. For our choice of tissue properties, variations of +50% and −50% in intracellular conductivities, respectively yield changes of +20% and −27.5% in transmural propagation speed, yet only of +1.7% and −4.6% for equivalent variations in the extracellular tensor, which therefore explains the minor contribution of the latter to the width of the QRS complex.

### Variability in body conductivities modulates QRS amplitudes

**Figure 4:**
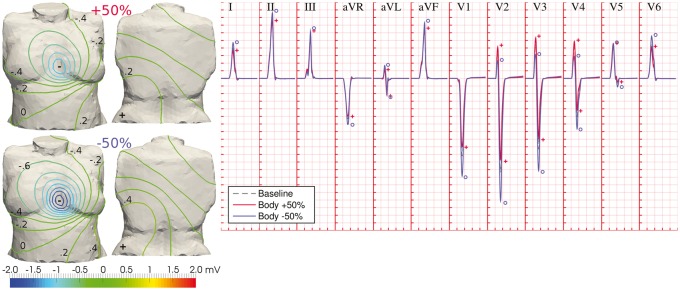
Variability in body conductivities modulates QRS amplitudes. Differences in the diffusion of electrical potential across the human torso affect the magnitudes of BSPs, without significantly altering their spatial pattern (left panel; BSP distributions shown at time for R-peak in lead II). This is translated in inversely proportional QRS amplitudes to body conductivities, and increased R-wave progression (right panel). Dashed grey ECG traces: baseline conditions; solid red and blue ECG traces: increased and decreased torso conductivity, respectively. Symbols indicate maximum wave amplitudes under variability in torso conductivity (same colour code). Simulated ECG grid resolution: 40 ms/0.1 mV.

Similar to the role of extracellular myocardial conductivities, variability in body conductivities (torso/lungs/bones) exerted a scaling effect on QRS amplitudes by affecting the magnitudes of BSPs. Results on the effects of this variability in torso conductivity are illustrated in *Figure **4*, as this exhibited the largest contribution in the human QRS complex compared to lung and bone conductivities (Supplementary material online, *Figure S2*).

Although the spatial patterns of BSPs remained mostly unaltered under variations of body conduction properties, the larger diffusion of extracellular potentials associated with larger conductivities yielded BSPs with lower magnitudes at times for R-peak, whilst smaller body conductivities accentuated the magnitude of the observed gradients (*Figure **4*, left panel). This was thus translated in inversely proportional QRS amplitudes to body conductivities in all leads (R-peak amplitudes of 92 ± 6% and 112 ± 10% of baseline for increased/decreased torso conductivities, respectively). In the precordial leads, reduced body conductivities increased R-wave progression (*Figure **4*, solid blue traces), and vice versa.

### Variability in location of endocardial root points affects S-wave progression

Additional simulation studies were performed in order to evaluate how anatomical variability in the location of endocardial activation root points (at coupling sites of the free-running Purkinje system) impacts the human QRS complex. Eight activation sequences were designed based on the anatomical variability reported on the human trifascicular system,^9^ as well as the early activation sites in the *exvivo* microelectrode studies by Durrer *et al.*^4^ and *invivo* electromechanical wave imaging by Provost *et al.*^5^ These included six activation sequences altering the position of LV anterior and posterior coupling sites towards more apical and basal locations, and two for more apical and/or lateral RV bundle coupling sites.

**Figure 5:**
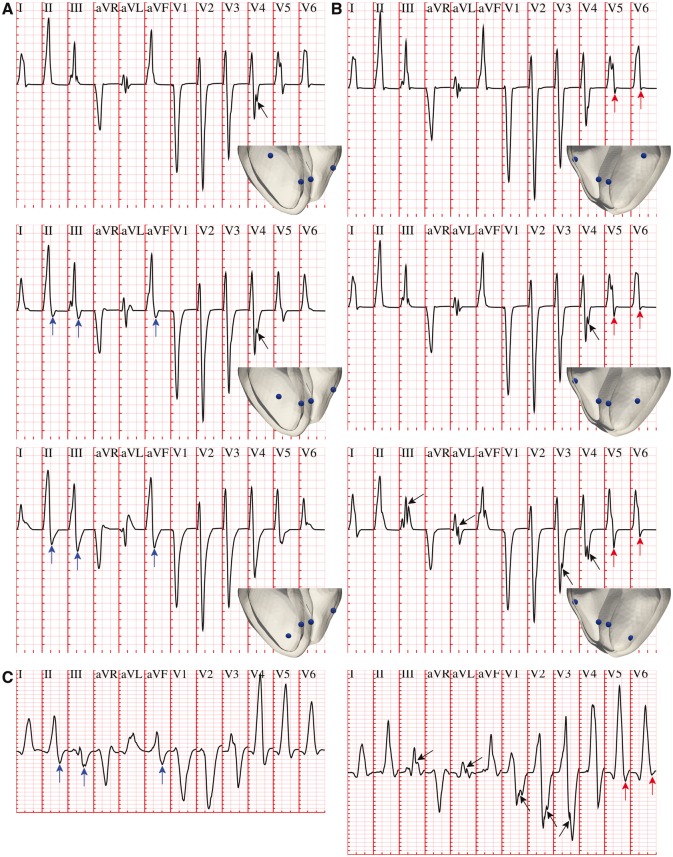
Variability in the location of LV endocardial activation sites affects S-wave progression. (*A*) More apical coupling sites of the LV anterior fascicular branch were associated with increasingly more negative S-waves in leads II, III and aVF (blue arrows). (*B*) Similar trends were present for a more basal coupling of the LV posterior fascicular branch in precordial leads V5 and V6 (red arrows). Variability in LV stimulation also resulted in notched QRS complexes in precordial and limb leads (black arrows). (*C*) QRS complexes of two healthy individuals in the PTB Diagnostic ECG Database^8^ (subjects 237 and 117, respectively), exhibiting similar QRS features. Simulated and clinical ECG grid resolutions: 40 ms/0.1 mV.

Variability in the location of early activation on the anterior LV wall, from more basal towards more apical positions (*Figure **5**A*, top to bottom), was associated with increasing S-wave progression in the limb and augmented limb leads (*Figure **5**A*, blue arrows). This progression was especially marked in leads II, III and aVF, exhibiting increasingly more negative S-waves. Variability in the apico-basal location of the LV posterior wall site was similarly correlated with increasing S-wave progression in precordial leads V5 and V6 (*Figure **5**B*, red arrows), as these are the only electrodes with a solid angle view of the LV posterior wall. Some of the activation sequences also presented notched QRS complexes in precordial and limb leads (*Figure **5**A and B*, black arrows), as a consequence of variability in the positions of simultaneous LV earliest epicardial breakthroughs. Such QRS features were also observed in healthy individuals of the PTB Diagnostic ECG Database, as shown in the two ECG excerpts presented in *Figure **5**C*. On the other hand, activation sequences with more apical and/or lateral RV sites led to similar QRS morphologies compared to baseline (Supplementary material online, *Figure S3*). Simultaneous variations of more than one LV/RV coupling sites led to an additive effect of the above discussed contributions.

### Knockout of root nodes reproduce QRS phenotype caused by intraventricular conduction defects

**Figure 6:**
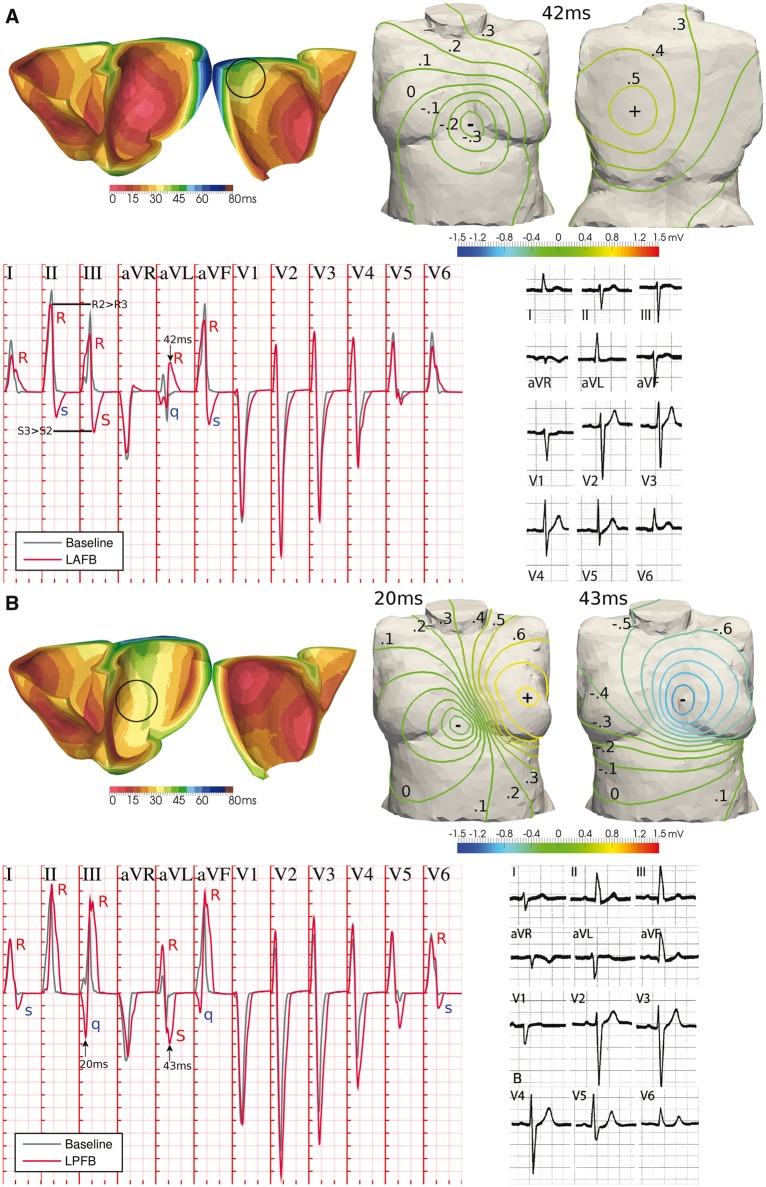
Hemiblock of the LV human conduction system. (*A*) Knockout of the LV anterior branch resulted in the late activation of the LV basal free wall, moving the positive pole in body surface potentials from the left hip to the centre of the back, and recovering the clinical manifestations of left anterior fascicular block (LAFB). (*B*) Similarly, LV posterior wall stimulation knockout resulted in delayed LV basal paraseptal activation, affecting the time course and position of the negative pole in the front of the torso, with the associated clinical manifestations of left posterior fascicular block (LPFB). Representative ECGs of LAFB and LPFB are reproduced with permission from Elizari *et al.*^9^ ECG grid resolutions: 40 ms/0.1 mV (simulation); 200 ms/0.5 mV (clinical).

**Figure 7:**
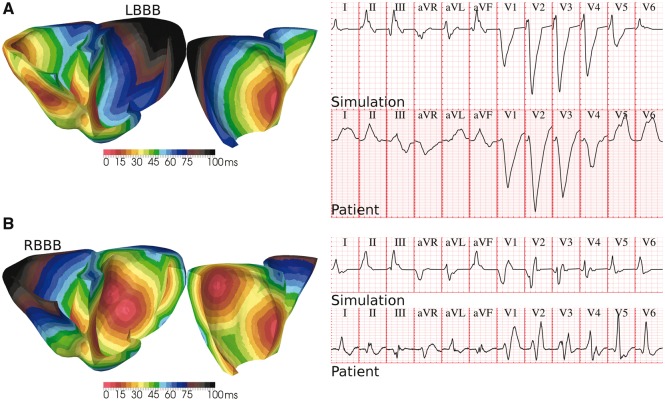
Bundle branch block of the human conduction system. (*A*) A lateral progression of activation in LV due to the simultaneous knockout of the LV anterior/posterior branches closely mimics the diagnostic criteria of left bundle branch block (LBBB) in precordial leads V1–V6. (*B*) Similar results for lateral progression of activation in RV, associated with right bundle branch block (RBBB). QRS complexes of LBBB and RBBB patients in the PTB Diagnostic ECG Database^8^ (subjects 208 and 209, respectively) are provided for comparison. Clinical and simulated ECG grids resolution: 40 ms/0.1 mV.

Due to their direct correspondence with the trifascicular activation system, variability in the number of root nodes can be used to reproduce pathophysiology of the human conduction system. This is illustrated in *Figures **6** and **7* for LV anterior/posterior hemiblocks and complete LV/RV bundle branch blocks, respectively.

Knocking out the activation of the LV anterior wall (*Figure **6**A*, black circle) resulted in the late activation of the LV basal free wall. While the negative pole on the chest was almost unvaried, this late region caused the positive pole in BSPs to move up from the left hip to the centre of the back, yielding a late QRS vector mainly oriented from front to rear torso surfaces and slightly leftwards and upwards. As a consequence, the reconstructed QRS complex exhibits the clinical manifestations of a left anterior fascicular block^9^ (LAFB; lower-right panel of *Figure **6**A*): QRS width smaller than 120 ms; left-deviated late QRS axis to −50°; qR pattern in aVL and RS/Rs patterns in leads II, III and aVF, with wave amplitudes satisfying RII > RIII and SIII > SII; and delayed intrinsicoid deflection (time from Q to R peaks) in aVL of 42 ms, close to the clinical threshold of 45 ms.

Similarly, the knockout of LV posterior wall activation (*Figure **6**B*, black circle) was associated with a delayed activation of the posterior LV basal wall, severely affecting the time course and position of the poles in BSPs on the chest. Such a distribution of BSPs recovered the clinical phenotype of a left posterior fascicular block^9^ (LPFB; lower-right panel of *Figure **6**B*): QRS width smaller than 120 ms; right-deviated late QRS axis to 100°; Rs or RS patterns in leads I and aVL; qR patterns in leads III and aVF; and S waves in all precordial leads, with intrinsicoid deflection times in V6 (24 ms) and aVF (34 ms) greater than in aVL (21 ms). The less-symmetric (trapezoidal-like) morphology in the last deflection of the QRS complex of all limb and augmented limb leads observed in the clinical recordings is also recovered.

Finally, a complete knockout of both LV anterior and posterior branches led to a lateral progression of LV activation, significantly delaying septal to basal LV stimulation (*Figure **7**A*). This retrieved the characteristic signature of left bundle branch block (LBBB) in precordial leads: QRS complex of at least 120 ms; marked QS pattern in V1; and notched R-wave in lead V6. Similarly, the knockout of coupling sites on the RV led to a lateral sequence of RV activation (*Figure **7**B*), yielding the manifestation of right bundle branch block (RBBB): QRS duration of at least 120 ms; terminal R-wave in lead V1; slurred S-waves in leads I and V6.

## Discussion

This study presents a detailed investigation on how variability in the main determinants of the human activation sequence and passive myocardial and body conduction properties translates into variability in clinical QRS biomarkers. Our approach tightly couples state-of-the-art anatomically-based multiscale bidomain simulations of human ventricular electrophysiology with *exvivo* and *invivo* human activation data. This allows augmenting the information attainable from scarce and limited experimental recordings in human (usually available for only small numbers of subjects, of low-resolution, and highly variable between individuals) and gaining novel physiological knowledge on the information enclosed in the human QRS complex.

R-peak amplitudes exhibit the largest amount of variability, as these are shown to be simultaneously modulated by endocardial activation speed, and by myocardial intracellular and extracellular conductivities. Variability in torso and organs conductivities shows an impact on QRS magnitude, in agreement with the literature.^13^ On the contrary, QRS width was only regulated by activation speed and intracellular myocardial conductivities, whereas intrinsicoid deflection (QR interval) is only affected by the endocardial activation. In particular, there is a high impact in reducing the activation sequence speed on QRS duration. These insights can be of clinical relevance in order to separate from the ECG the contribution of these three factors, as well as to make progress towards more personalized activation sequences in computational studies.

Variability in the anatomical locations of activation root sites (corresponding to the coupling of the LV fascicular bundles with the myocardium) was associated with S-wave progression in the limb and precordial leads, and occasional notched QRS complexes in the precordial derivations. Whereas insights in S-wave progression could be used to further particularize patient-specific activation sequences, it is important to remark that the notched QRS complexes occurred in the absence of structural defects in the ventricles, in spite of their frequent association. Finally, variability in the number of activation sites recovered the pathological landmarks introduced by disease in the human conduction system in the QRS complex, both under partial and complete bundle block conditions.

Different modelling approaches have been used to date to study the ventricular activation sequence on the human ECG. These vary in generality, from specifying activation times analytically by means of a parameterized sequence,^15^ to the creation of idealized Purkinje networks including explicit Purkinje-muscle junctions.^16^^,^^17^ They also differ in computational complexity, from the semi-automatic generation of activation profiles,^10^^,^^18^ to more iterative and interactive parametrization processes to converge on a personalized activation sequence.^16^ The activation sequences utilized here encapsulate the minimal information needed to investigate the implication on the QRS complex of anatomical variability in the human trifascicular conduction system. This allows for an accurate, robust, and efficient simulation of the human QRS complex, requiring minimum user input beyond the specification of Purkinje-branch end points.

Compared to previous studies, our work represents a detailed investigation on how variability at the conduction level (progression of the activation sequence, passive tissue conductivities, and anatomical location of Purkinje-myocardial coupling) impacts the variability observed at the human QRS complex. Although in this work we have explicitly concentrated our investigations in exploring variability at the tissue level, the tools presented here could be easily combined with experimentally-calibrated populations of models to capture human variability at the cellular electrophysiological level.^12^ This may considerably advance the field of computational cardiac electrophysiology towards a more predictive technology for risk assessment at the population level, as both variability in conduction and cellular electrophysiology modulate individual responses to pharmacological and electrical therapy.

An accurate modelling of pathological conditions of the human activation system might also yield a better understanding of clinical ECG biomarkers of disease. Our activation sequences have been shown to replicate the main diagnostic manifestations of left anterior fascicular, left posterior fascicular, left bundle branch and right bundle branch block conditions. Previous computational studies have also shown agreement in diseased QRS biomarkers associated to left bundle branch block, using either a similar modelling approach to ours for the human activation sequence,^18^ or more detailed anatomical representations of the human Purkinje network.^16^ Sahli *et al.*^17^ also addressed the modelling of right bundle branch block using detailed Purkinje trees, however, without probing its impact on the precordial leads, which are the derivations used in its clinical diagnosis. To the best of our knowledge, modelling of fascicular hemiblocks on the human QRS complex has not been previously addressed in the literature. The adoption of the activation sequences proposed here could therefore also promote the computational modelling of other pathological conditions (myocardial infarction, ischaemia, heart failure, cardiomyopathies), frequently affected by comorbidities in the human conduction system.

Variability in the location of activation sites from our baseline configuration had only a small influence on the QRS axis. This may explain the preponderance of a QRS axis around 60° in healthy individuals, as reflected by the PTB Diagnostic ECG Database^8^ where 65% (33/51) of the healthy cohort was classified as having QRS axis between 30° and 70°. It is known that a vertical heart orientation (tall, thin individuals) is manifested in a right QRS axis shift, whilst a left QRS axis is exhibited under more horizontal heart orientations (short, broad individuals).^9^ The simulation results, therefore, reveal that the location of stimulation sites and the rest of varied parameters cannot explain the large variability in the healthy QRS axis observed clinically. Therefore, other factors such as the relative orientation of the human heart within the thoracic cavity, ventricular mass and torso geometry might be the main determinants of variability in the healthy human QRS complex.^19^ Testing of these hypotheses would require the expensive generation of new heart-torso anatomical models with variable heart and torso volumes and ventricular orientation, and will therefore be addressed in future investigations. Corrections to account for this geometrical variability have been proposed,^19^ which will also be investigated in future work.

The activation sequences presented here have been shown to be a feasible approach to capture the main intraventricular conduction defects of the human conduction system. However, their underlying simplifications may limit their applicability to model other pathophysiological contributions of the Purkinje system.^20^ This may include ventricular extrasystoles arising from Purkinje fibres in idiopathic intrafascicular tachycardia, or retrograde propagation through the Purkinje network. Whereas the former could *a priori* be easily addressed by timing additional ectopic foci in conjunction with the default activation sequence, the latter is likely to require a full coupling of the ventricles with a detailed anatomical representation of the Purkinje system. In addition, our results have not been compared against those obtained by using more detailed anatomical representations of the Purkinje system, due to the lack of high-resolution data in human in which to build such models. This may constitute an interesting line of future research shall the experimental data in human becomes available, as well as an in-depth comparison against different tissue descriptions of myocardial conduction.

**Conflict of interest**: none declared.

## Funding

L. Cardone-Noott was supported by the Engineering and Physical Sciences Research Council (EP/G03706X/1). B. Rodriguez, A. Bueno-Orovio and A. Mincholé were supported by B. Rodriguez’ Welcome Trust Senior Research Fellowship in Basic Biomedical Science (100246/Z/12/Z) and the British Heart Foundation Centre of Research Excellence (RE/13/1/30181).

## Supplementary Material

Supplementary Data
