# Supplementary material for: Human ventricular activation sequence and the simulation of the electrocardiographic QRS complex and its variability in healthy and intraventricular block conditions
Source: Europace. 2016 Dec 23;18(Suppl 4):iv4–iv15. doi: 10.1093/europace/euw346 (PMC5225966; doi:10.1093/europace/euw346)
Supplement: Supplementary Data [file euw346_supp.pdf]

Human ventricular activation sequence and the simulation of the electrocardiographic QRS complex and its variability in healthy and intraventricular block conditions

Louie Cardone-Noott, Alfonso Bueno-Orovio, Ana Mincholé, Nejib Zemzemi, Blanca Rodriguez

SUPPLEMENTARY METHODS

Mesh information and tissue properties

The biventricular mesh used in this work was generated from a multi-slice computer tomography scan as previously described[1], with an edge length of 0.4 mm, and contains 2.51 million nodes and 14.2 million tetrahedral elements. The biventricular mesh was embedded in a torso volume containing lung and bone regions, generated from computer tomography images of a 43 year old woman. The heart and torso meshes were combined by rotating and translating into anatomically-realistic concordance. The combined heart-torso mesh consists of about 3.25 million nodes and 19.4 million tetrahedra.

Myocardial fibre structure was generated using a rule-based method to replicate the findings of Streeter *et al.*[2] Tissue conductivities were chosen to generate realistic conduction velocities for the choice of cell model, numerical scheme, and mesh resolution. For the myocardium, the three orthotropic intracellular conductivities were 1.5, 0.45, and 0.225 mS/cm in the fibre, sheet, and inter-sheet directions, respectively. Axisymmetric extracellular conductivities of 5.46, 2.03 and 2.03 mS/cm were calculated from these intracellular values scaled by measured resistivity ratios[3]. In a 1D fibre model these conductivities resulted in conduction velocities of 57.0, 25.9, 16.2 cm/s, close to the 67, 30, and 17 cm/s wave speeds reported in pig[4]. Isotropic conductivities of 0.389 and 0.2 mS/cm were assigned to the lung and bone elements[5], and 2.16 mS/cm in the rest of the torso[6].

As no regional gradients have been reported in the healthy human ventricles in the fast sodium current, which is the main current sustaining ventricular activation, we used the endocardial variant of the 2006 ten Tusscher-Panfilov model[7] throughout the entire ventricles for model simplification.

Numerical solver

The full coupled heart-torso model was solved simultaneously for improved accuracy and

convergence[8]. All simulations were solved using the finite element solver Chaste[9] with a variable ODE time step and a bidomain PDE time step of 25 μs. The convergence and accuracy of our electrophysiological solver has already been demonstrated during activation for our choice of mesh resolution, time discretisation and cellular model[10]. A 150 ms simulation typically required 30 minutes of computer time on 240 CPU cores. Post-processing of simulation results was performed using custom scripts in ParaView and MATLAB.

SUPPLEMENTARY FIGURE LEGENDS

Figure S1. Visualisation of the combined heart-torso mesh. The coloured spheres indicate the location of the virtual electrodes, using standard (European) colour-coding.

Figure S2. Effects of variability in lung and bone conductivities on QRS amplitudes. **A:** Lung conductivity. **B:** Bone conductivity. Dashed grey ECG traces: baseline conditions; solid red and blue ECG traces: increased and decreased conductivities, respectively. ECG grid resolution: 40 ms/0.1 mV.

Figure S3. Variability in the location of RV free-running Purkinje coupling sites. **A:** More apical RV anterior activation site. **B:** More apical RV posterior activation site. ECG grid resolution: 40 ms/0.1 mV.

SUPPLEMENTARY REFERENCES

1. Sebastian R, Ordas S, Plank G, Rodriguez B, Vigmond EJ, Frangi AF. Assessing influence of conductivity in heart modelling with the aim of studying cardiovascular diseases. *Proc. SPIE 6916, Medical Imaging 2008: Physiology, Function, and Structure from Medical Images* 2008. p. 691627–10.

2. Streeter DD, Spotnitz HM, Patel DP, Ross J, Sonnenblick EH. Fiber Orientation in the Canine Left Ventricle during Diastole and Systole. *Circ Res* 1969;**24**:339–47.

3. Clerc L. Directional differences of impulse spread in trabecular muscle from mammalian heart. *J Physiol* 1976;**255**:335–46.

4. Caldwell BJ, Trew ML, Sands GB, Hooks DA, LeGrice IJ, Smaill BH. Three distinct directions of intramural activation reveal nonuniform side-to-side electrical coupling of ventricular myocytes. *Circ Arrhythm Electrophysiol* 2009;**2**:433–40.

5. Gabriel C, Gabriel S, Corthout E. The dielectric properties of biological tissues: I. Literature survey. *Phys Med Biol* 1996;**41**:2231–49.

6. Rush S, Abildskov JA, McFeer R. Resistivity of body tissues at low frequencies. *Circ Res* 1963;**12**:40–50.

7. Tusscher KHWJ ten, Panfilov AV. Alternans and spiral breakup in a human ventricular tissue model. *Am J Physiol Heart Circ Physiol* 2006;**291**:H1088–100.

8. Zemzemi N, Bernabeu MO, Saiz J, Cooper J, Pathmanathan P, Mirams GR, *et al.* Computational assessment of drug-induced effects on the electrocardiogram: from ion channel to body surface potentials. *Br J Pharmacol* 2013;**168**:718–33.

9. Pitt-Francis J, Pathmanathan P, Bernabeu MO, Bordas R, Cooper J, Fletcher AG, *et al.* Chaste: A test-driven approach to software development for biological modelling. *Comput Phys Commun* 2009;**180**:2452–71.

10. Niederer SA, Kerfoot E, Benson AP, Bernabeu MO, Bernus O, Bradley C, *et al.* Verification of cardiac tissue electrophysiology simulators using an N-version benchmark. *Philos Trans R Soc Math Phys Eng Sci* 2011;**369**:4331–51.

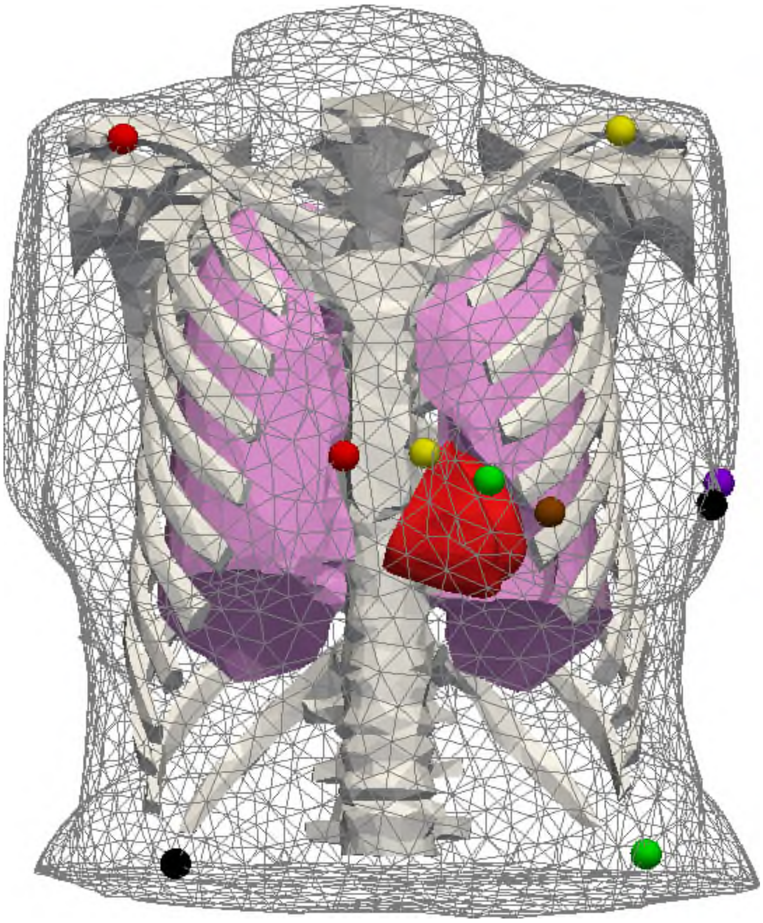

Supplementary Figure S1

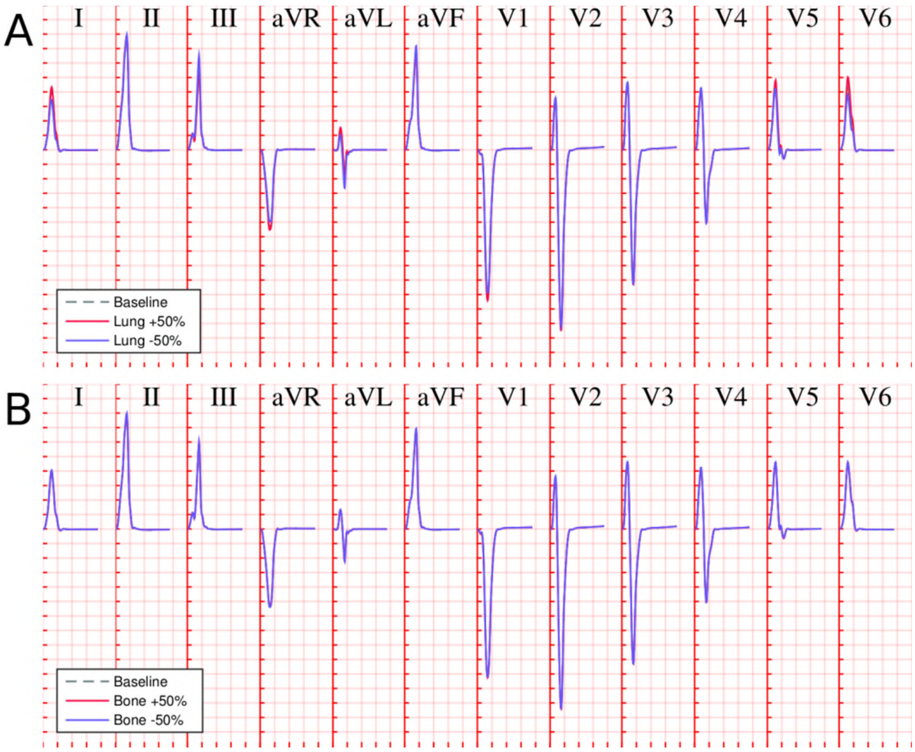

Supplementary Figure S2

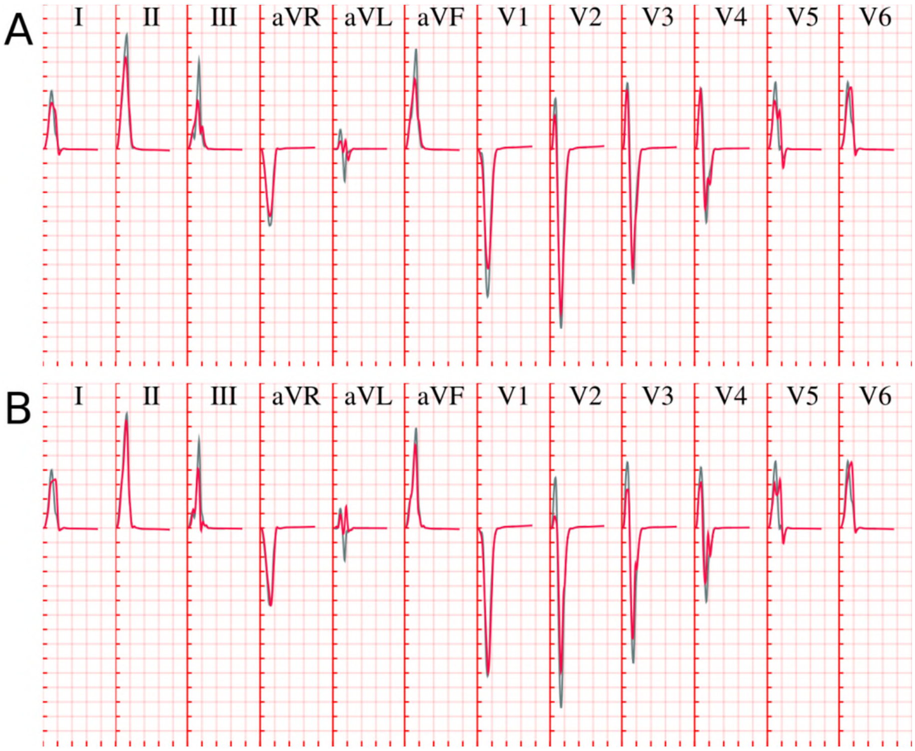

Supplementary Figure S3
